# Influence of temperature management at 33 °C versus normothermia on survival in patients with vasopressor support after out-of-hospital cardiac arrest: a post hoc analysis of the TTM-2 trial

**DOI:** 10.1186/s13054-022-04107-9

**Published:** 2022-07-31

**Authors:** Joachim Düring, Martin Annborn, Alain Cariou, Michelle S. Chew, Josef Dankiewicz, Hans Friberg, Matthias Haenggi, Zana Haxhija, Janus C. Jakobsen, Halvor Langeland, Fabio Silvio Taccone, Matthew Thomas, Susann Ullén, Matt P. Wise, Niklas Nielsen

**Affiliations:** 1 Department of Clinical Sciences, Anesthesia and Intensive Care, Lund University, Skåne University Hospital, Malmö, Sweden; 2 Department of Clinical Sciences, Anesthesia and Intensive Care, Lund University, Helsingborg Hospital, Helsingborg, Sweden; 3 Cochin University Hospital (APHP) and University of Paris (Medical School), Paris, France; 4 Department of Anesthesia and Intensive Care, Biomedical and Clinical Sciences, Linköping University, Linköping, Sweden; 5 Department of Clinical Sciences, Cardiology, Lund University, Skåne University Hospital, Lund, Sweden; 6 Department of Intensive Care Medicine, Inselspital, Bern University Hospital, University of Bern, Bern, Switzerland; 7 Copenhagen Trial Unit, Centre for Clinical Intervention Research, Capital Region of Denmark, Denmark; 8 Department of Regional Health Research, The Faculty of Health Sciences, University of Southern Denmark, Odense, Denmark; 9 Department of Anesthesiology and Intensive Care Medicine, St. Olav’s University Hospital, Trondheim, Norway; 10 Institute of Circulation and Medical Imaging, Faculty of Medicine and Health Sciences, NTNU, Trondheim, Norway; 11 Department of Intensive Care, Hôpital Erasme, Université Libre de Bruxelles, Brussels, Belgium; 12 Department of Intensive Care, University Hospitals Bristol and Weston, Bristol, UK; 13 Clinical Studies Sweden- Forum South, Skåne University Hospital, Lund, Sweden; 14 Adult Critical Care, University Hospital of Wales, Cardiff, UK

**Keywords:** Heart arrest, Cardiac arrest, Sudden, Hypothermia induced, Shock, Mortality

## Abstract

**Background:**

Targeted temperature management at 33 °C (TTM33) has been employed in effort to mitigate brain injury in unconscious survivors of out-of-hospital cardiac arrest (OHCA). Current guidelines recommend prevention of fever, not excluding TTM33. The main objective of this study was to investigate if TTM33 is associated with mortality in patients with vasopressor support on admission after OHCA.

**Methods:**

We performed a post hoc analysis of patients included in the TTM-2 trial, an international, multicenter trial, investigating outcomes in unconscious adult OHCA patients randomized to TTM33 versus normothermia. Patients were grouped according to level of circulatory support on admission: (1) no-vasopressor support, mean arterial blood pressure (MAP) ≥ 70 mmHg; (2) moderate-vasopressor support MAP < 70 mmHg or any dose of dopamine/dobutamine or noradrenaline/adrenaline dose ≤ 0.25 µg/kg/min; and (3) high-vasopressor support, noradrenaline/adrenaline dose > 0.25 µg/kg/min. Hazard ratios with TTM33 were calculated for all-cause 180-day mortality in these groups.

**Results:**

The TTM-2 trial enrolled 1900 patients. Data on primary outcome were available for 1850 patients, with 662, 896, and 292 patients in the, no-, moderate-, or high-vasopressor support groups, respectively. Hazard ratio for 180-day mortality was 1.04 [98.3% CI 0.78–1.39] in the no-, 1.22 [98.3% CI 0.97–1.53] in the moderate-, and 0.97 [98.3% CI 0.68–1.38] in the high-vasopressor support groups with regard to TTM33. Results were consistent in an imputed, adjusted sensitivity analysis.

**Conclusions:**

In this exploratory analysis, temperature control at 33 °C after OHCA, compared to normothermia, was not associated with higher incidence of death in patients stratified according to vasopressor support on admission.

*Trial registration* Clinical trials identifier NCT02908308, registered September 20, 2016.

**Supplementary Information:**

The online version contains supplementary material available at 10.1186/s13054-022-04107-9.

## Background

Induced hypothermia for neurologic protection after cardiac arrest has been established in experimental animal models [1]. Further, targeted temperature management (TTM) at a core body temperature of 33 °C (TTM33) has been a key component in post-cardiac arrest care for unconscious survivors after out-of-hospital cardiac arrest (OHCA) since initial randomized trials [2, 3] suggested improved outcome. Later trials [4, 5], however, have been unable to reproduce the initial positive results. Current guidelines recommend avoidance of fever, defined as a core body temperature above 37.7 °C, not excluding TTM33 [6]. Using TTM33 might have clinically relevant cardiovascular effects, such as increased vasoconstriction and reduced cardiac index, without a reduction in mixed venous saturation [7]. In the TTM-trial [4], in patients with shock on admission, the hemodynamic alterations caused by hypothermia/rewarming induced increased circulatory failure and were associated with a non-significant increase in ICU mortality [8]; however, the TTM-2 trial did not show any increased risk of mortality in OHCA patients with shock on admission [5]. Our hypothesis is that potential deleterious cardiovascular effects, of targeted temperature management at 33 °C, have minimal impact in patients with no cardiovascular failure (resilience) or major cardiovascular failure (established injuries to vital organ systems), but could influence circulatory outcomes for patients with marginal cardiovascular status without established injuries (at risk population). ILCOR states that it is unknown whether certain subpopulation might benefit from temperature management at a lower or higher target [9]. We therefore performed an exploratory post hoc analysis of patients included in the TTM-2 trial [5], with the primary objective to investigate any association of a temperature intervention in patients with early vasopressor support on all-cause mortality after OHCA. Secondary objectives included exploration of the association between temperature intervention and modes of death in patients with different levels of cardiovascular support.

## Methods

### Trial design

This is an exploratory *post hoc* analysis of the “Hypothermia versus Normothermia after Out-of-Hospital Cardiac Arrest” trial (TTM-2 trial) [5], an international, investigator-initiated open-label superiority trial investigating the efficacy of targeted hypothermia versus targeted normothermia with early treatment of fever on mortality. The design [10] and statistical plan [11] of the TTM2 trial have been previously published. The protocol was approved by the ethics committees in each participating country. Written informed consent was waived, deferred, or obtained from a legal surrogate, depending on the circumstances, and was obtained from each patient who regained mental capacity.

### Participants

Adult (≥ 18 years of age), unconscious (not able to obey verbal commands and no verbal response to pain) survivors of OHCA of presumed cardiac or unknown cause were screened consecutively. Eligible patients had sustained return of spontaneous circulation (ROSC), defined as more than 20 consecutive minutes of palpable pulses, without the need for chest compressions. The main exclusion criteria were an interval from return of spontaneous circulation to screening of more than 180 min, unwitnessed cardiac arrest with asystole as the initial rhythm, or limitations in care.

### Randomization and blinding

Eligible patients were randomized in a 1:1 ratio to hypothermia or normothermia, stratified by site, using a web-based system with permutated blocks of varying sizes. Health care providers involved in patient care were aware of intervention allocation, whereas the physicians performing neurologic prognostication and study administrators were blinded.

### Interventions

Patients assigned to hypothermia were immediately cooled and maintained at a core body temperature of 33 °C, until 28 h after randomization, after which temperature was increased by 0.33 °C per hour. Core body temperature was continuously monitored in patients randomized to normothermia, and no active cooling or warming was provided unless the patient developed a core body temperature ≥ 37.8 °C, at which time active cooling to 37.5 °C was started. Total duration of the intervention was 40 h in both intervention groups, and sedation was mandatory during this period. Following the intervention, unconscious patients in both allocation arms developing fever, temperature ≥ 37.8 °C, were actively kept at a body temperature of 37.5 °C until able to follow verbal command or up to 72 h after randomization.

### Neurologic prognostication and withdrawal of life-sustaining therapy

Neurologic prognosis was assessed by a physician blinded to trial intervention at 96 h according to a neurological prognostication protocol. Withdrawal of life-sustaining therapies was at the discretion of the treating physician, but was not to be initiated based on presumed poor neurologic prognosis prior to neurological prognostication.

### Definitions of vasopressor support on admission

We categorized patients according to circulatory characteristics on admission (Additional file 1: Fig. S1), into three subgroups: (1) no-vasopressor support (No-VS), mean arterial blood pressure (MAP) ≥ 70 with no inotropic or vasopressor support; (2) moderate-vasopressor support (Moderate-VS), MAP < 70 or any dose dopamine, or dobutamine, or noradrenaline/adrenaline dose ≤ 0.25 µg/kg/min; and (3) high-vasopressor support (High-VS), noradrenaline/adrenaline dose > 0.25 µg/kg/min.

### Outcomes

The primary outcome in this study was all-cause mortality at 180 days after randomization. Secondary outcomes included: (1) cause of death, categorized as neurological or non-neurological for patients that died within 30 days. Death cause was based on the subjective assessment of the bedside healthcare team; (2) high-vasopressor requirement, categorized as > 0.25 µg/kg/min of noradrenaline/adrenaline or dead in the ICU Day 1–4; (3–5) heart rate, mean arterial pressure, and lactate between baseline and 72 h; and (6) the occurrence of hemodynamically compromising arrhythmia.

### Statistics

Descriptive statistics are presented as medians with interquartile range and frequencies, respectively. Differences in baseline variables were assessed using Chi-square test or Mann–Whitney test as appropriate. Unadjusted Cox regression was used to estimate survival probability censored at 180 days, and cumulative risk of neurological/non-neurological death censored at 30 days between intervention groups. A Cox regression model with multiple imputations was used as sensitivity analysis for both all-cause mortality and cause of death (Additional file 1: Figs. S2, S3). Results from Cox regression models are presented as hazard ratios (HR) with Bonferroni-adjusted confidence intervals (CI), as were confidence intervals for repeated measurements. The odds ratio (OR) for highest daily vasopressor dose was estimated using unadjusted logistic regression. Model fit was assessed using the Hosmer–Lemeshow test. Heart rate, mean arterial pressure, and lactate are presented as medians with interquartile range, and differences between intervention groups are presented as difference between medians using the percentile bootstrap method.

## Results

The TTM2 trial enrolled 1900 patients between November 2017 and January 2020. Consent could not be obtained or was withdrawn in 37 patients, 2 patients were randomized twice, leading to a study population of 1861 subjects, 931 in the hypothermia group and 930 patients in the normothermia group. In the study population, 666 patients (36%) had No-VS, 902 (48%) Moderate-VS, and 293 (16%) High-VS on admission. Minor imbalances were detected between study groups regarding previous cerebrovascular disease, previous cardiac disease, administered bystander CPR and admission pH (Table 1).

**Table 1:** Baseline characteristics of the study population

	No-vasopressor support		Moderate-vasopressor support		High-vasopressor support	
	TTM33	Normothermia	TTM33	Normothermia	TTM33	Normothermia
*n*	346	320	448	454	136	157
Age (median [IQR])	63 [54, 72]	62 [52, 71]	67 [59, 75]	65 [57, 74]	66 [58, 74]	68 [58, 75]
Male sex (%)	286 (83)	264 (83)	353 (79)	353 (78)	103 (76)	118 (75)
*Comorbidity*						
Cardiac disease (%)	177 (53)	135 (44)	256 (61)	248 (58)	67 (51)	80 (54)
Coronary artery disease (%)	69 (21)	60 (20)	112 (27)	115 (27)	29 (22)	35 (24)
Diabetes (%)	59 (17)	45 (14)	87 (19)	94 (21)	27 (20)	28 (18)
Renal disease (%)	13 (4)	14 (4)	21 (5)	19 (4)	9 (7)	16 (10)
COPD (%)	30 (9)	23 (7)	53 (12)	47 (10)	23 (17)	23 (15)
Cerebrovascular disease (%)	19 (6)	15 (5)	42 (9)	23 (5)	8 (6)	13 (8)
Liver disease (%)	3 (1)	0 (0)	5 (1)	2 (0.4)	3 (2)	1 (0.6)
Frailty score ≥ 4 (%)	69 (20)	58 (18)	104 (23)	117 (26)	37 (27)	46 (29)
*Peri-arrest characteristics*						
Cardiac cause of arrest (%)	308 (89)	289 (90)	406 (91)	395 (87)	110 (81)	130 (83)
AMI (%)	160 (47)	152 (48)	215 (49)	207 (46)	63 (47)	78 (50)
Witnessed arrest (%)	317 (92)	293 (92)	407 (91)	409 (90)	126 (93)	150 (96)
Bystander CPR performed (%)	270 (78)	267 (83)	380 (85)	352 (78)	109 (80)	109 (69)
Shockable rhythm (%)	264 (78)	262 (83)	327 (75)	333 (75)	80 (60)	105 (70)
Minutes to ALS (median [IQR])	10 [5, 15]	9 [6, 13]	10 [6, 15]	10 [6, 14]	10 [5, 16]	10 [5, 16]
Defibrillations (median [IQR])	2 [1, 4]	2 [1, 4]	2 [1, 4]	2 [1, 4]	2 [0, 5]	2 [1, 5]
Adrenaline, mg (median [IQR])	1 [0, 2]	1 [0, 2]	2 [0, 3]	2 [0, 3]	3 [1, 5]	2 [1, 4]
Minutes to ROSC (median [IQR])	23 [15, 35]	22 [15, 34]	26 [17, 40]	25 [17, 40]	34 [20, 53]	32 [20, 49]
*Circulatory status on admission (%)*						
MAP ≥ 70 mmHg	346 (100)	320 (100)	NA	NA	NA	NA
MAP < 70 mmHg	0 (0)	0 (0)	96 (21)	101 (22)	NA	NA
Dopamine or dobutamine	0 (0)	0 (0)	12 (3)	20 (4)	NA	NA
Adr/noradr ≤ 0.1 µg/kg/min	0 (0)	0 (0)	199 (44)	181 (40)	0 (0)	0 (0)
Adr/noradr 0.10–0.25 µg/kg/min	0 (0)	0 (0)	141 (32)	152 (34)	0 (0)	0 (0)
Adr/noradr > 0.25 µg/kg/min	0 (0)	0 (0)	0 (0)	0 (0)	136 (100)	157 (100)
Lactate, mmol/l (median [IQR])	4.2 [2.3, 6.6]	4.2 [2.2, 7.1]	4.9 [2.6, 8.0]	4.7 [2.4, 8.0]	7.9 [4.7, 10.5]	6.8 [3.7, 10.4]
pH (median [IQR])	7.24 [7.16, 7.30]	7.24 [7.15, 7.30]	7.22 [7.11, 7.30]	7.21 [7.10, 7.28]	7.11 [6.98, 7.22]	7.16 [7.02, 7.26]

Baseline characteristics for the TTM2 intention-to-treat population divided in subgroups of circulatory support on admission and stratified according to intervention

Adr, Adrenaline; ALS, Advanced life support; AMI, Acute myocardial infarction, defined as ST-elevation or new onset left bundle branch block on admission electrocardiogram; COPD, Chronic obstructive pulmonary disease; CPR, Cardiopulmonary resuscitation; ROSC, Return of spontaneous circulation; MAP, Mean arterial pressure; Noradr, Noradrenaline; and TTM33, Targeted temperature management at 33 °C

### Mortality

**Fig. 1:**
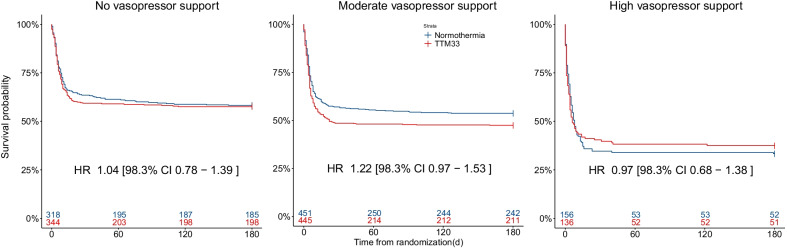
Probability of survival. Kaplan–Meier graph censored at 180 days indicating probability of survival in subgroups of vasopressor support on admission, stratified according to temperature intervention. No-vasopressor support, mean arterial blood pressure (MAP) ≥ 70 with no inotropic or vasopressor support; moderate-vasopressor support, MAP < 70 or any dose dopamine, or dobutamine, or noradrenaline/adrenaline dose ≤ 0.25 µg/kg/min; and high-vasopressor support, noradrenaline/adrenaline dose > 0.25 µg/kg/min. Colored numbers at bottom of plot illustrate number of patients at risk in respective strata at specified timepoint. The vertical tick-marks correspond to censored data. Hazard ratios (HR) are presented with 95% confidence intervals; TTM33, targeted temperature management at 33 °C

Data on 180-day survival were missing in 11 subjects, 4 (0.6%) in the subgroup with No-VS at ICU admission, 6 (0.7%) in the Moderate-VS group, and 1 (0.3%) in the High-VS group. In a pooled population analysis, the incidence of death was higher in patients with Moderate-VS, HR 1.30 [95% CI 1.12–1.51], and High-VS 2.12 [95% CI 1.76–2.55] when compared to the No-VS group (Additional file 1: Fig. S1). At 180 days, mortality in the Moderate-VS group was 53% for TTM33 and 46% for patients treated with normothermia. The hazard ratio (HR) for 180-day all-cause mortality in patients randomized to TTM33 when compared to normothermia was: HR 1.04 [98.3% CI 0.78–1.39] in the No-VS group; HR 1.22 [98.3% CI 0.97–1.53] in the Moderate-VS group; and HR 0.97 [98.3% CI 0.68–1.38] in the High-VS group (Fig. 1). The interaction of Moderate-VS on admission with TTM33 was associated with a tendency toward higher incidence of death, HR 1.47 [98.3% CI 1.00–2.17] in the sensitivity analysis (Additional file 1: Fig. S2).

### Cause of death

**Fig. 2:**
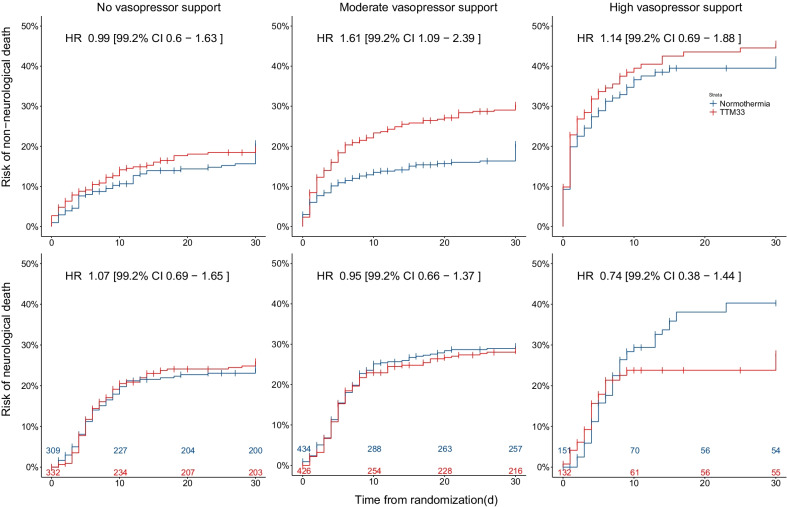
Cause of death. Kaplan–Meier graph censored at 30 days indicating cumulative risk of non-neurological versus neurological mortality in subgroups of vasopressor support on admission, stratified according to temperature intervention. No-vasopressor support, mean arterial blood pressure (MAP) ≥ 70 with no inotropic or vasopressor support; moderate-vasopressor support, MAP < 70 or any dose dopamine, or dobutamine, or noradrenaline/adrenaline dose ≤ 0.25 µg/kg/min; and high-vasopressor support, noradrenaline/adrenaline dose > 0.25 µg/kg/min. Colored numbers at bottom of plot illustrate number of patients at risk in respective strata at specified timepoint. The vertical tick-marks correspond to censored data. Hazard ratios (HR) are presented with 95% confidence intervals; TTM33, targeted temperature management at 33 °C

The cause of death was available for 1784 patients (96%). In the Moderate-VS group (*n* = 860), 115 (27%) patients treated with TTM33 and 77 (18%) patients treated with normothermia died within 30 days from a non-neurological cause, HR 1.61 [99.2% CI 1.09–2.39] (Fig. 2), and results were similar in sensitivity analysis (Additional file 1: Fig. S3). No significant differences were detected for death modality in the No-VS and High-VS groups.

### High-vasopressor requirement day 1–4

**Fig. 3:**
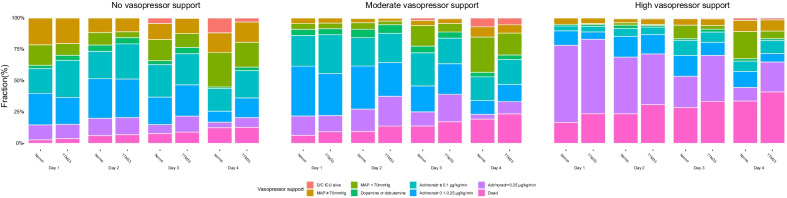
Circulatory status day 1–4. Graph illustrating the distribution of highest recorded circulatory support for each day. Patients are categorized according to vasopressor support on admission and stratified according to temperature intervention. No-vasopressor support, mean arterial blood pressure (MAP) ≥ 70 with no inotropic or vasopressor support; moderate-vasopressor support, MAP < 70 or any dose dopamine, or dobutamine, or noradrenaline/adrenaline dose ≤ 0.25 µg/kg/min; and high-vasopressor support, noradrenaline/adrenaline dose > 0.25 µg/kg/min. D/C, Discharge; ICU, Intensive care unit; Normo; normothermia; and TTM33, targeted temperature management at 33 °C

TTM33 was associated with a higher odds for high-vasopressor requirement in the Moderate-VS group: OR 1.60 [99% CI 1.11–2.32]; 1.92 [99% CI 1.32–2.80]; and 1.66 [99% CI 1.13–2.45] on day two, three, and four, respectively. Similar findings were found for the High-VS group on day three and four, while no association with intervention was found in the No-VS group (Fig. 3).

### Heart rate

**Fig. 4:**
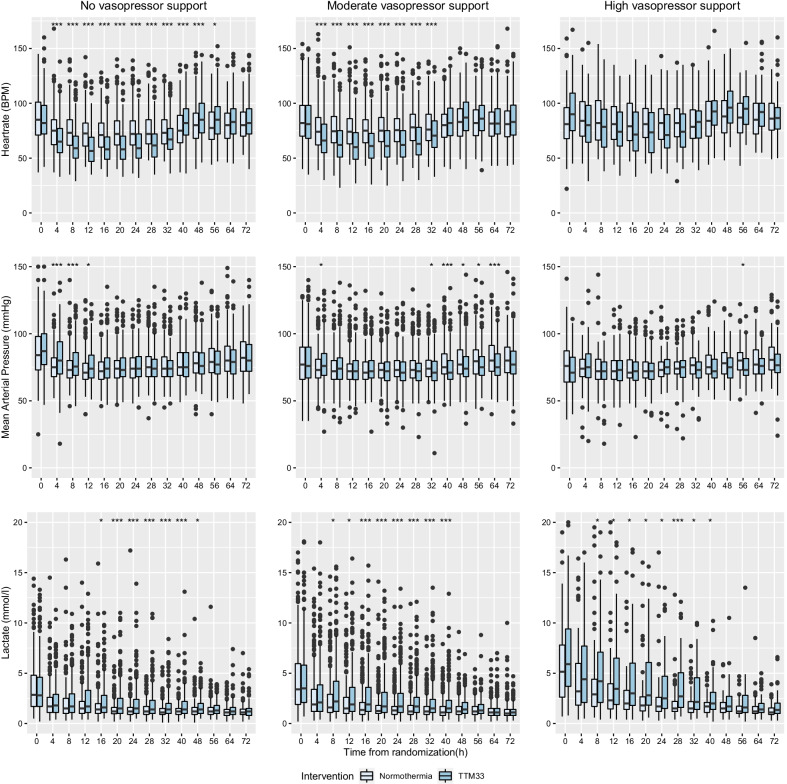
Hemodynamics 0–72 h. Heart rate, mean arterial pressure, and lactate during the 0–72 h after randomization in groups of different levels of circulatory support on admission and stratified by temperature intervention at 33 °C versus normothermia. No-vasopressor support, mean arterial blood pressure (MAP) ≥ 70 with no inotropic or vasopressor support; moderate-vasopressor support, MAP < 70 or any dose dopamine, or dobutamine, or noradrenaline/adrenaline dose ≤ 0.25 µg/kg/min; and high-vasopressor support, noradrenaline/adrenaline dose > 0.25 µg/kg/min. Boxes represent the interquartile range (IQR), with medians marked as vertical bands. Whiskers symbolize 1.5 × IQR, and dots outside this range represent outliers. TTM33; Targeted temperature management at 33 °C. *p < 0.003; **p < 0.0001; ***p < 0.00001

Patients with No-VS randomized to TTM33 had a lower heart rate at 4–32 h, with a maximum median difference of − 16 [99.7% CI − 21 to − 9] beats per minute (BPM) at 12 h, and higher heart rate at 40–56 h, with a max difference of 8 [99.7% CI 1–13] BPM at 56 h. The subgroup with Moderate-VS randomized to TTM33 had lower heart rate at 4–32 h, with a maximum difference at 28 h − 15 [99.7% CI − 18 to − 9] BPM. No significant difference in heart rate was detected during the 0–72 h in the group with High-VS (Fig. 4, Additional file 1: Fig. S4).

### Mean arterial pressure

In the subgroup with No-VS on admission, TTM33 compared to normothermia was associated with higher mean arterial blood pressure at 4–12 h with a maximum median difference between intervention groups of 5 mmHg [99.7% CI 0–9] at 4 h. In the subgroup with Moderate-VS, TTM33 was associated with increased MAP at 4 h, 3 mmHg [99.7% CI 0–6] and lower MAP at 32–64 h, with maximum at difference between intervention groups at 64 h, MAP − 5 [99.7% CI − 8 to − 1] mmHg. Patients treated with TTM33 in the subgroup with High-VS had lower MAP at 56 h, MAP − 7 mmHg [99.7% CI − 11 to − 2] (Fig. 4, Additional file 1: Fig. S5).

### Lactate

TTM33 was associated with higher level of lactate; No-VS at 16–48 h, maximum difference of 0.3 [99.7% CI 0.1–0.5] mmol/l at 24 h, Moderate-VS at 8–40 h, max 0.6 [99.7% CI 0.1–1.1] mmol/l at 8 h, High-VS at 8–40 h, maximum difference of 0.6 [99.7% CI 0.0–1.0] mmol/l at 8 h (Fig. 4, Additional file 1: Fig. S6).

### Hemodynamically compromising arrhythmia

The risk ratio for hemodynamically compromising arrhythmia in patients treated with TTM33 was: 1.05 [98.3% CI 0.77–1.44] in the Moderate-VS group and 1.31 [98.3% CI 0.88–1.96] in the High-VS group, compared to No-VS group.

## Discussion

The major findings in this exploratory post hoc analysis of the TTM-2 trial are that unconscious OHCA patients with moderate-vasopressor support on admission, receiving TTM at 33 °C, had an increased incidence of non-neurological death, while no significant difference in 180-day all-cause mortality rate was detected in any of the vasopressor support groups.

The effect of temperature management in the context of severity of the post-cardiac arrest syndrome (PCAS) has not been investigated in any randomized trial, but observational reports suggest that there may be a differential impact on outcome according to level of PCAS [12–14]. Four previous trials have randomized unconscious OHCA patients to a temperature intervention below 34 °C versus normothermia [2, 3, 5, 15], of which only one reported outcomes for patients stratified by hemodynamic status on admission. In the predefined subgroup analysis of the TTM-2 trial [5], the relative risk of death with an intervention of TTM33 among patients in shock on admission (*n* = 534) was reported to be non-statistically significant. Shock on admission was defined as a systolic blood pressure of less than 90 mmHg for more than 30 min or the need for supportive measure to maintain a systolic ≥ 90 mmHg and end‐organ hypoperfusion (cool arms and legs, urine output < 30 ml per hour, and heart rate < 60 beats per minute). Similar to the results of the predefined subgroup analysis of the TTM-2 trial, a post hoc subgroup analysis consisting of 139 patients in shock on admission in the TTM-trial [4], comparing TTM33 versus TTM at 36 °C (TTM36), found no association between the temperature interventions and incidence of death, censored at 180 days [8]. Vasopressor support on admission was more frequent in our analysis than the frequency of shock on admission in the TTM and TTM-2 trial, which can be explained by slightly different definitions. In a study of 435 OHCA patients stratified according to serum lactate on admission in three strata, the subgroup with the most severe hyperlactatemia, ≥ 12 mmol/l, a temperature intervention below 35 °C was associated with more favorable neurologic outcome in an adjusted analysis [13]. In a similar study [14] of 1111 patients, stratified into three subgroups according to the revised post-Cardiac Arrest Syndrome for Therapeutic hypothermia scoring system (rCAST), grading the severity of PCAS (including initial rhythm, lactate, pH, neurologic status and time to ROSC), a temperature intervention below 35 °C was associated with higher survival at 30 days in adjusted analysis in the group with moderate-level rCAST, but not in the other groups. The results of these two Japanese registry studies of a highly selected patient population are in contrast to our findings. The design and stratification of these studies make comparisons with our analyses difficult. In a single-center observational study of 911 cardiac arrest patients without signs of severe brain injury, TTM36 was associated with improved hospital survival in a subgroup of patients with noradrenaline/adrenaline dose < 0.1 µg/kg/min, while TTM33 was associated with higher hospital survival in patents with vasopressor doses ≥ 0.1 µg/kg/min [12]. These results are not in line with our primary analysis; however, subgroup stratification and interventions were different.

In our study, TTM33 was associated with higher dose vasopressor therapy during the ICU stay in the groups with vasopressor support on admission, indicating increased hemodynamic compromise with TTM33. The TTM2 trial reported an increased incidence of hemodynamically compromising arrhythmia with TTM33 [5], but this does not seem to explain the increased incidence of non-neurological death, as the relative risk is similar in the subgroups of our analysis. Previously, temperature management at 33 °C has been associated with increased systemic vascular resistance, lactate levels, and decreased cardiac output due to decreased heart rate and stroke volume [7]. It has been suggested that cardiovascular frail patients fare better without TTM33 [8], presumably due to hypothermia induced increased systemic vascular resistance in combination with vasopressor therapy leading to decompensated congestive heart failure, reduced cardiac output, and end-organ hypoperfusion.

Heart rate decreased during hypothermia in No-/Moderate-VS, but increased after hypothermia only in the No-VS group. Differences in lactate levels between intervention groups, however, were minor in vasopressor support groups, making decreased cardiac output less plausible to account for the additional mortality. Findings in this study suggesting that patients with Moderate-VS/High-VS were more vulnerable to the additional TTM33 were: (1) less increase in blood pressure in response to induction of hypothermia in subgroups with Moderate-VS/High-VS; (2) patients with Moderate-VS/High-VS treated with TTM33 had lower blood pressure after rewarming, while no such association was detected in the group with No-VS; (3) rewarming was associated with an increase in heart rate for patients with TTM33, only in the No-VS group; and (4) the association of high dose vasopressor requirement, with TTM33 coincided with rewarming in the Moderate-VS group, and persisted after normalization of body temperature. The higher vasopressor doses did not correspond to a higher MAP among patients in the Moderate-VS/High-VS groups, and blood pressure was lower with induced hypothermia/rewarming in these groups. These findings may suggest excess rate of non-neurological death attributed to relative hypotension in the Moderate-VS group, caused by a hypothermia acquired inability to increase heart rate in combination with vasodilatation triggered by rewarming, and augmented by a cytokine response [16]. Incidence of non-neurological death in the High-VS group, however, did not increase. This could be due to low statistical power or support our hypothesis that these patients, to a higher degree, have already sustained injuries to vital organ systems that are not further aggravated by the circulatory deterioration of induced hypothermia with subsequent rewarming. The increased rate of short term non-neurological mortality in our study did not influence all-cause mortality at 6 months at a statistically significant level, possibly due to competing risk of severe cerebral injuries in this population.

Our results are the first to suggest potential circulatory harm in a subgroup of unconscious survivors of cardiac arrest from a temperature intervention at 33 °C. We propose that future temperature intervention studies, in the context of cardiac arrest, should include predefined subgroup analyses for patients with different circulatory states.

### Limitations

Our exploratory results should be interpreted cautiously in the context of a post hoc subgroup analysis, as we risk introducing bias. Hemodynamic data used for categorization of patients, based on the severity of cardiovascular failure, were only available in the form of extended cardiovascular SOFA score (ordinal data), introducing risk of spurious findings. Also, the data available did not allow us to adjust for multiple vasopressors. The primary analysis was carried out on three subgroups, on the basis that the deleterious hemodynamic effects had a threshold and a ceiling effect. Our subsequent analyses show patients with No-VS to be resilient to potential deleterious effects of TTM33 on circulatory status, and patients with Moderate-VS/High-VS to be vulnerable to the impact of TTM33 on hemodynamics. Whether a ceiling effect exists cannot be definitively concluded from our data. Cause of death was based on subjective assessments of clinical data. Interrater agreement has, however, been reported as substantial in a study including five different death modalities [17]. These results stem from a large randomized trial in well-sourced health care systems, which vouch for the external validity in similar systems, but not necessarily in a resource challenged environment.

## Conclusion

In this exploratory analysis, temperature control at 33 °C after OHCA, compared to normothermia and early treatment of fever, was not associated with higher mortality ratio in patients stratified according to vasopressor support at admission.

## Supplementary Information

**Additional file 1:** Additional Figures S1–S6.

## Data Availability

The data that support the findings of this study were used under license for the current study and so are not publicly available.

## Abbreviations

BPM: Beats per minute
CI: Confidence interval
High-VS: High-vasopressor support at admission
HR: Hazard rate
ICU: Intensive care unit
MAP: Mean arterial blood pressure
Moderate-VS: Moderate-vasopressor support at admission
No-VS: No-vasopressor support at admission
OHCA: Out-of-hospital cardiac arrest
PCAS: Post-cardiac arrest syndrome
RR: Relative risk
SD: Standard deviation
TTM: Targeted temperature management
TTM33: Targeted temperature management at 33 °C
