# Supplementary material for: Influence of temperature management at 33 °C versus normothermia on survival in patients with vasopressor support after out-of-hospital cardiac arrest: a post hoc analysis of the TTM-2 trial
Source: Crit Care. 2022 Jul 31;26:231. doi: 10.1186/s13054-022-04107-9 (PMC9339193; doi:10.1186/s13054-022-04107-9)
Supplement: Supplementary file 1 — Additional file 1: Additional Figures S1–S6. [file 13054_2022_4107_MOESM1_ESM.docx]

**Additional file 1**

Influence of temperature management at 33ºC versus normothermia on survival in patients with vasopressor support after out-of-hospital cardiac arrest: A post-hoc analysis of the TTM-2 trial

Joachim Düring^1^, Martin Annborn^2^, Alain Cariou^3^, Michelle S. Chew^4^, Josef Dankiewicz^5^, Hans Friberg^1^, Matthias Haenggi^6^, Zana Haxhija^1^, Janus C. Jakobsen^7,8^, Halvor Langeland^9,10^, Fabio Silvio Taccone^11^, Matthew Thomas^12^, Susann Ullén^13^, Matt P. Wise^14^, Niklas Nielsen^2^

^1 1^ Department of Clinical Sciences, Anesthesia & Intensive care, Lund University, Skåne University Hospital, Malmö, Sweden.

^2^ Department of Clinical Sciences Lund, Anesthesia & Intensive care, Lund University, Helsingborg Hospital, Helsingborg, Sweden.

^3^ Cochin University Hospital (APHP) and University of Paris (Medical School), Paris, France

^4^ Department of Anesthesia and Intensive Care, Biomedical and Clinical Sciences, Linköping University, Linköping, Sweden

^5^ Department of Clinical Sciences, Cardiology, Lund University, Skåne University Hospital, Lund, Sweden.

^6^ Department of Intensive Care Medicine, Inselspital, Bern University Hospital, University of Bern, Switzerland

^7^ Copenhagen Trial Unit, Centre for Clinical Intervention Research, Capital region of Denmark, Denmark

^8^ Department of Regional Health Research, The Faculty of Health Sciences, University of Southern Denmark, Denmark

^9^ Department of Anesthesiology and Intensive Care Medicine, St. Olav's University Hospital, Trondheim, Norway

^10^ Institute of Circulation and Medical Imaging, Faculty of Medicine and Health Sciences, NTNU, Trondheim, Norway

^11^ Department of Intensive Care, Hôpital Erasme, Université Libre de Bruxelles, Brussels, Belgium.

^12^ Department of Intensive Care, University Hospitals Bristol and Weston, Bristol, UK

^13^ Clinical Studies Sweden- Forum South, Skåne University Hospital, Lund

^14^ Adult Critical Care, University Hospital of Wales, Cardiff, UK

*Rationale for categorization of vasopressor support on admission*

In the TTM2-trial, categorization of circulatory characteristics on admission was based on the extended cardiovascular Sequential Organ Failure Assessment [1], and adapted within the constraints of the electronic case report form used in the TTM2-trial. Our hypothesis is that potential deleterious circulatory effects, of induced hypothermia with subsequent rewarming, have minimal impact in patients with no circulatory failure (resilience) or major circulatory failure (established injuries to vital organ systems), but could influence outcomes for patients with marginal circulatory status without established injuries (at risk population). We categorized patients into three subgroups according to circulatory status on admission. In lack of published consensus of what constitutes high vasopressor support in post cardiac arrest care, cutoffs were chosen based on the lowest vasopressor dose causing separation of 180-day all-cause mortality for all three groups (Additional File 1 Figure 1), based on all randomized patients. In an observational study, vasopressin used as an adjunctive measure in septic shock with high vasopressor support [2], is started at our chosen cutoff of noradrenaline/adrenaline > 0.25 µg/kg/min, supporting our categorization.

Vasopressor support vs probability of survival

**Additional file 1: Figure S1.** Survival probability after cardiac arrest censored at 180 days for the TTM2 population stratified according to circulatory status on admission based on all randomized patients. Hazard ratios (HR) are presented with 95% confidence intervals. Mean arterial pressure (MAP) ≥ 70 mmHg with no vasopressor/inotropes as reference category; HR, Hazard Ratio; Noradr, Noradrenaline; Adr, Adrenaline.

*Statistical methodology for sensitivity analysis*

A Cox regression model with multiple imputations was used as sensitivity analysis. Model was adjusted for: age, sex, location of arrest (place of residency, yes/no), time to return of spontaneous circulation, witnessed arrest, bystander CPR preformed, dose of adrenaline, shockable rhythm, previous cardiac disease, previous cerebrovascular disease, pH on admission, TTM33, Vasopressor support on admission (reference category No-VS), and the interaction between TTM33 and vasopressor support on admission (reference group No-VS and normothermia). The multiple imputation was performed using chained equations with predictive mean matching for continuous variables and logistic regression for categorical data[3]. Outcome and all adjustors in model were used for imputation. Estimates were based on pooled estimates from 20 datasets, using Rubins rules. Imputation was performed under the assumption of missing at random. Skewed continuous explanatory variables were transformed to normal distribution, choosing the method yielding the lowest Pearson P statistic/degrees of freedom, scaled to standard deviations and centered. For the cox regression models the proportional hazards and linearity assumptions were confirmed by inspecting Schoenfield and Martingale residuals. No influential observations were detected when inspecting residual deviance of model variables. Variance inflation factor for variables in model did not infer any non-structural multicollinearity issues.

Sensitivity analysis all-cause mortality rate

**Additional file 1: Figure S2.** Forest plot illustrating the hazard ratio for all-cause mortality censored at 180 days after randomization, in an adjusted Cox regression model with multiple imputations. Hazard ratios are presented with 98.3 % confidence intervals. Reference level for targeted temperature management at 33ºC (TTM33) is normothermia, and for vasopressor support levels, no vasopressor support on admission. Moderate-VS, moderate vasopressor support, MAP < 70 or any dose dopamine, or dobutamine, or noradrenaline/adrenaline dose ≤ 0.25 µg/kg/min; High-VS, high vasopressor support, noradrenaline/adrenaline dose > 0.25 µg/kg/min; SD, standard deviation.

Sensitivity analysis incidence of non-neurological etiology of death

**Additional file 1: Figure S3.** Forest plot illustrating the hazard ratio for non-neurological death censored at 30 days after randomization, in an adjusted Cox regression model with multiple imputations. Hazard ratios are presented with 99.2 % confidence intervals. Reference level for targeted temperature management at 33ºC (TTM33) is normothermia, and for vasopressor support levels, no vasopressor support on admission. Moderate-VS, moderate vasopressor support, MAP < 70 or any dose dopamine, or dobutamine, or noradrenaline/adrenaline dose ≤ 0.25 µg/kg/min; High-VS, high vasopressor support, noradrenaline/adrenaline dose > 0.25 µg/kg/min; SD, standard deviation.

Heart rate

**Additional file 1: Figure S4.** Heart rate in the TTM2 population, stratified according to intervention. The median difference in heart rate 28 h and 40 h after randomization was -13[-17 to - 9], p<0.00001, 5 beats per minute [1- 9], p<0.00001, respectively in patients treated with targeted temperature management at 33ºC (TTM33), versus patients treated with normothermia. Boxes represents the interquartile range (IQR), with medians marked as vertical bands. Whiskers symbolize 1.5 x IQR, and dots outside this range represent outliers. * p<0.003, ** p<0.0001, *** p<0.00001.

Mean arterial pressure

**Additional file 1: Figure S5.** Mean arterial pressure in the TTM2 population, stratified according to intervention. The median difference in mean arterial pressure 28 h and 40 h after randomization was 0 [-2 - 2], p=0.21, -2 mmHg [-5 to -0], p<0.0002, respectively in patients treated with targeted temperature management at 33ºC (TTM33), versus patients treated with normothermia. Boxes represents the interquartile range (IQR), with medians marked as vertical bands. Whiskers symbolize 1.5 x IQR, and dots outside this range represent outliers. * p<0.003, ** p<0.0001, *** p<0.00001.

Lactate concentration

**Additional file 1: Figure S6.** Lactate concentration in the TTM2 population, stratified according to intervention. The median difference in lactate concentration 28 h and 40 h after randomization was 0.4 [0.2-0.6], p<0.00001 and 0.3 mmol/l [0.1-0.5], p<0.00001, respectively in patients treated with targeted temperature management at 33ºC (TTM33), versus patients treated with normothermia. Boxes represents the interquartile range (IQR), with medians marked as vertical bands. Whiskers symbolize 1.5 x IQR, and dots outside this range represent outliers. * p<0.003, ** p<0.0001, *** p<0.00001.

**References**

1. Annborn M, Bro-Jeppesen J, Nielsen N, Ullen S, Kjaergaard J, Hassager C, Wanscher M, Hovdenes J, Pellis T, Pelosi P *et al*: **The association of targeted temperature management at 33 and 36 degrees C with outcome in patients with moderate shock on admission after out-of-hospital cardiac arrest: a post hoc analysis of the Target Temperature Management trial**. *Intensive Care Med* 2014, **40**(9):1210-1219.

2. Sacha GL, Lam SW, Wang L, Duggal A, Reddy AJ, Bauer SR: **Association of Catecholamine Dose, Lactate, and Shock Duration at Vasopressin Initiation With Mortality in Patients With Septic Shock**. *Crit Care Med* 2021.

3. van Buuren S, Groothuis-Oudshoorn K: **mice: Multivariate Imputation by Chained Equations in R**. *2011* 2011, **45**(3):67.
